# Neuroprotective Effect of Sonic Hedgehog Mediated PI3K/AKT Pathway in Amyotrophic Lateral Sclerosis Model Mice

**DOI:** 10.1007/s12035-022-03013-z

**Published:** 2022-09-03

**Authors:** Yan Qi, Chen Yang, Hui Zhao, Zhanjin Deng, Jin Xu, Weijing Liang, Zhitang Sun, John Dirk Vestergaard Nieland

**Affiliations:** 1 Department of Neurology, Second Hospital of Shanxi Medical University, Shanxi, China; 2 Shanxi Medical University, Shanxi, China; 3 Department of Health Science and Technology, Aalborg University, 9220 Aalborg, Denmark

**Keywords:** ALS, PI3K/AKT signaling pathway, SHH, Gli-1, SOD1-G93A

## Abstract

The Sonic Hedgehog (SHH) signaling pathway is related to the progression of various tumors and nervous system diseases. Still, its specific role in neurodegenerative diseases, such as amyotrophic lateral sclerosis (ALS), remains studied. This research investigates the role of SHH and PI3K/AKT signaling pathway proteins on ALS development in a SOD1-G93A transgenic mouse model. After injection of SHH and PI3K/AKT signaling pathway inhibitors or agonists in hSOD1-G93A (9 weeks of age) transgenic mice, we studied skeletal muscle pathology using immunohistochemical staining and Western blot methods. In addition, recorded data on rotation time, weight, and survival were analyzed for these mice. Our study showed that the expression of SHH, Gli-1 and p-AKT in ALS mice decreased with the progression of the disease. The expression of p-AKT changed together with Gli-1 while injecting PI3K/AKT signaling pathway inhibitor or agonist; SHH and Gli-1 protein expression remained unchanged; p-AKT protein expression significantly decreased while injecting PI3K/AKT signaling pathway inhibitor. These results indicate that SHH has a regulatory effect on PI3K/AKT signaling pathway. In behavioral experiments, we found that the survival time of hSOD1-G93A mice was prolonged by injection of SHH agonist while shortened by injection of SHH inhibitor. In conclusion, we confirmed that the SHH pathway played a neuroprotective role in ALS by mediating PI3K/AKT signaling pathway.

## Introduction

Amyotrophic lateral sclerosis (ALS) is a neurodegenerative disease. It is the most common motor neuron disease. The patient eventually dies because of the loss of the upper and lower motor neurons that control voluntary movement. Moreover, the average course of the disease is only 3–5 years. So far, there is no effective treatment for ALS [1]. The disease is a complex disease caused by multiple factors and genes. These pathogenic theories have been more widely accepted: glutamate excitotoxicity, oxidative stress, protein misfolding, mitochondrial changes, axon transport disorders, neuroinflammation, exogenous toxins, virus infection, gene mutation, abnormal RNA metabolism, and lipid metabolism [2–7]. The superoxide dismutase 1 (SOD1) gene is one of the main pathogenic genes of ALS. Since SOD1 mutation was found, more than 30 genes are associated with familial Amyotrophic Lateral Sclerosis (fALS). Among these genes, five (C9ORF72, SOD1, TARDBP, FUS, TBK1) had the highest mutation frequency in fALS, which were 40%, 20%, 5%, < 5% and < 5% respectively [8]. Abnormal expression of SOD1 can produce toxicity, cause oxidative stress damage, and affect the structure and function of normal neurons [9].

Lee et al. found the hedgehog (HH) gene for the first time in the study of wingless mutation in Drosophila [10]. The HH family of spinal animals is divided into three subgroups, namely Desert Hedgehog (DHH), India hedgehog (IHH), and Sonic Hedgehog (SHH). SHH is widely expressed in human tissues [11]. The SHH signaling pathway mainly comprises the membrane receptors patched (Ptch1), smoothened (SMO), and the downstream transcription factors Gli family. The Gli family of transcription factors includes Gli1, Gli2, and Gli3 [12, 13]. In the presence of SHH, Gli1 plays a significant role in activating the pathway to be used as an indicator of the expression of this pathway [14]. The SHH signaling pathway is essential for vertebrate embryonic development, dynamic tissue balance, and regeneration [15]. It is an essential signaling pathway regarding the formation of the nervous system [16]. Studies have shown that the SHH pathway can play multiple protective effects on acute brain injury, long-term memory impairment, and synaptic damage [17]. The imbalance of SHH gene expression may restrict the development and growth of the forebrain and hypothalamus ventral midline and even result in craniofacial deformity [18]. Studies have shown that SHH gene expression can improve cell survival by increasing cell proliferation and promoting the G1 cell cycle [19]. SHH signal transduction is crucial to regulating apoptosis [20]. At the same time, the activation of the SHH signaling pathway is related to the progress of various tumors [21] and nervous system diseases [22].

In recent years, studies have found that PI3K/AKT signaling pathway is involved in ALS disease, and neuroprotective drugs can exert a protective role through this pathway. For example, butylphthalide can activate PI3K/AKT/GSK-3β signal pathway in the ischemic cerebral infarction model, thereby reducing nerve function damage and protecting local nerve cells [23]. Improving mitochondrial dysfunction [24], inhibiting apoptosis, and reducing oxidative stress have been considered therapeutic strategies for ALS [25]. Many studies have shown that PI3K/AKT pathway can be activated to increase anti-apoptotic protein expression levels and reduce pro-apoptotic protein expression levels to improve cell survival rate and mitochondrial function in ALS. For example, Xiang Yin [26] and other studies have found that astrocyte elevation gene-1 (AEG-1) can regulate PI3K/AKT pathway. The lack of AEG-1 in ALS motor neurons leads to inhibition of the PI3K/AKT pathway and an increase in cell apoptosis. However, activation of the PI3K/AKT signaling pathway can protect the cerebral cortex and astrocytes [27] from the damage due to oxidative stress. Zhao et al. [28] found that SHH and PI3K/AKT signaling pathways are closely related to Neuro-2a cells apoptosis.

Based on the above studies, we selected the SOD1-G93A transgenic mouse model of ALS, and the study aimed to clarify the connection between SHH and PI3K/AKT signaling pathway and its role in ALS. To confirm that activation of the SHH signaling pathway can play a neuroprotective role by mediating the PI3K/AKT signaling pathway, we administered agonists and inhibitors of different signaling pathways. We analyzed disease phenotypes, muscle pathology, and behavioral manifestations in mice. This study provides a theoretical basis for the SHH pathway to be an effective target in ALS treatment.

## Materials and Methods

### Animals

Transgenic mice (C57B6.CgTg SOD1-G93A-1Gur/J) were originally from the Jackson Laboratory and later supplied by Professor Honglin Feng, Harbin Medical University, China. Stable breeding was maintained by mating purchased heterozygous SOD1-G93A male mice with C57BL/6 J female mice. The mice were kept in a temperature-controlled (22–24℃) room with a 12 h light and 12 h dark cycle providing clean drinking water and granular mouse feed, four per cage with a male and female ratio of 1:3. All animal protocols were reviewed and approved by the Institutional Animal Care and Use Committee of Shanxi Medical University (DW2022031).

hSOD1G93A-positive and age-matched negative mice were euthanized. The elected mice were anesthetized and perfused transcardially with phosphate-buffered saline (PBS, PH = 7.4) for 10 min, then 4% paraformaldehyde (PFA) in 0.1% PBS for 1 h. The spinal cord was dissected, post-fixed with 4% PFA for 48 h and then paraffin-embedded. A series of spinal cord sections were made and sectioned at a thickness of 5 μm. Muscle tissue was quickly removed from mouse gastrocnemius, then placed in liquid nitrogen and stored in a refrigerator.

#### QRT-PCR

In this study, to minimize the used number and reduce the pain of animals, mice carrying SOD1G93A mutations were identified by PCR amplification of the DNA extracted from the tails using protocols provided by Jackson Lab. The primers used were 5'-CTAGGCCACAGAATTGAAAGATCT-3' and 5'-GTAGGTGGAAATTCTAGCATCATCC-3' for IL-2; 5'-CATCAGCCCTAATCCATCTGA-3' and 5'-CGCGACTAACAATCAAAGTGA-3' for hSOD1. The mice DNA was extracted and added to the prepared PCR amplification system. We performed PCR using the SYBR® Green PCR Master Mix (Life Tech) on an ABI 7300 (Applied Biosystems, Foster City, CA, USA) with the following program: 95℃ for 5 min, once in total; 95℃ for 45 s, 58℃ for the 30 s, 72℃ for 30 s, 35 reaction cycles; 72℃ for 5 min, once in total; 4℃ for preservation. After electrophoresis, the gel was taken into the Imaging System (Bio-Rad, USA) and observed, photographed, and analyzed.

### Groups and Drug Treatments

Accumulating evidences [29–33] demonstrated that female mice show less variability in the survival time than male mice, and the instability of the males' endocrine hormones would affect the experimental results [34–36]. Therefore consistent with previous reports, female mice were elected in this study. Well-identified ALS females (9 weeks of age) were selected into ALS, ALS + PUR, ALS + CYC, ALS + LY, and WT littermates as a control group with ten experimental animals in each group. Wild-type (WT) SOD1 mice are negative littermates of the G93A-SOD1 mice in the same nest. Drugs were injected every three days from early-onset (9 weeks of age) until the behavioral end-stage of mice and finally removed spinal cord tissue after euthanasia. If the mice died unexpectedly during the experiment, the corresponding mouse-age mice were added as described above.

Purmorphamine (PUR) (15 mg/kg) activates SHH signaling pathway by binding and activating the SMO protein [12, 13, 37, 38]. Cyclopamine (CYC) (12 mg/kg) [39, 40] is an inhibitor of the SHH signaling pathway. LY294002 (10 mg/kg) [41] can inhibit the activation of the PI3K/Akt signaling pathway.

We dissolved the reagent in dimethyl sulfoxide (DMSO) and then diluted the mixture with pure water to 10 mg/mL (DMSO concentration of 5%). PUR (15 mg/kg), CYC (12 mg/kg), or LY294002 (10 mg/kg) was injected intraperitoneally. The control groups simultaneously received the intraperitoneal injection of the same equivalent solvent (5% DMSO concentration).

### Western Blotting

Fresh lumbar spinal cord tissue was extracted and put into RIPA lysate (AR0102-100, Boster), adding protease inhibitor PMSF(AR1178, Boster) and phosphatase inhibitor (AR1183, Boster). BCA protein concentration assay kit (AR0146, Boster) was used to determine the protein concentration.

The total proteins from the spinal cord were separated by electrophoresis using 10% SDS–polyacrylamide gel, then transferred onto PVDF membranes. The membranes were blocked with 5% non-fat milk for 2 h at room temperature, then incubated overnight at 4 °C with the following antibodies: Rabbit Anti-SHH Polyclonal Antibody (bs-1544R, Bioss), Rabbit Anti-Gli-1 Polyclonal Antibody (bs-1206R, Bioss), Phospho-AKT (Ser473, Cell Signaling Technology), AKT polyclonal antibody (AP0095, Bioworld) or β-actin (AP0060, Bioworld) antibodies. After washing, the membranes were incubated with Goat anti-Rabbit IgG (BA1054, Boster) for 1 h at room temperature. The blots were visualized by the chemiluminescence-based detection kit (ECL Kit, Boster). Densitometry analysis was performed with a System Gel Doc XR + IMAGE LAB (Bio-Rad, USA) and quantified with NIH Image software (Image J).

### Immunohistochemistry

To quantify the number of motor neurons, every 10th section was selected, with a total of 30 sections being collected for each animal. The horizontal sections of the mouse lumbar spinal cord were sectioned at a thickness of 5 μm. After gradient dewaxing and dehydration, all sections were subsequently rinsed three times with 0.1 M PBS and blocked in BSA of 5% for 20 min, then incubated 1 h at 4℃ with the following primary antibodies, anti-SHH, anti-Gli1, Phospho-AKT. Then the sections were incubated 30 min at 37℃ with Goat anti-Rabbit IgG and subsequently rinsed three times with 0.1 M PBS. SABC was added and incubated at 37℃ for 30 min, then rinsed. The specimens that developed color at room temperature with DAB were examined under a microscope. The samples were stained with Hematoxylin and finally sealed with resin. The expression of SHH, Gli1 and p-AKT cells in the cervical spinal cord was evaluated by Image J.

### Pathological Analysis of the Gastrocnemius Muscle

Fresh gastrocnemius was dissected and processed by liquid nitrogen freezing at a − 20℃ cryostat Section (10 μm). And it was stained by hematoxylin and eosin (H&E), nicotinamide adenine dinucleotide hydrogen tetrazolium reductase (NADH-TR), Oil red O, PAS staining, or Modified Gomori Trichrome (MGT). They were observed and photographed with a microscope (Motic VM). We calculate at the same magnification.

H&E: hematoxylin–eosin staining kit (C0105M, Beyotime); NADH-TR: NADH (ST358, Beyotime); Oil red O: Oil red O staining kit (C0157M, Beyotime); PAS staining: periodate Schiff staining (PAS) kit (C0142M, Beyotime); MGT: made by myself with phosphotungstic acid (0.6 g), glacial acetic acid (1.0 ml), solid green (0.3 g), chronotropic acid 2R (0.6 g), Distillation-Distillation water to 100 ml.

### Assessment of Mouse Motor Ability, Body Weight, and Survival

The dyskinesia of mice was evaluated by the 4-point scoring system [42]:

- 4 points for no signs of dyskinesia
- 3 points for noticeable shaking of hind limbs
- 2 points for abnormal gait
- 1 point for dragging at least one hind limb
- 0 point for cannot turn over in 30 s (0 point represents the end stage of the disease)

We calculated the survival rate of mice in different groups and recorded the survival time of mice. The bodyweight of mice was measured and recorded once a week from the initial stage of onset (9 weeks old) until the end of the mice. The criteria of an early stage of mice include lifting the tail of mice, hind limb tremor, and (or) limb weakness. The criteria of late-stage: the mice were placed in supine or lateral position (bilateral) and could not turn over within 30 s.

Rotarod test was performed from 63 days of age once a week to evaluate the motor ability of ALS mice. Mice were trained at 8 rpm for a week before performing a formal Rotarod Test. We recorded the time of the mice on the rotating rod instrument with a diameter of 3 cm (16 rpm). The maximum recorded value is 180 s, but it is recorded as the actual time when it is lower than 180 s.

### Statistical Analysis

We designed graphs with Graph Pad Prism 8. Data were displayed as mean ± standard deviation. Statistical analysis was carried out with a t-test and one-way ANOVA. Repeated measurement data ANOVA was adopted to analyze the body weight and rotation time data. Moreover, the Kaplan Meier life test performed the probability of survival. Differences were statistically significant at *P* < 0.05.

## Results

### SHH and PI3K/AKT Pathways are Involved in the Development of ALS Disease

The SOD1 G93A female (ALS) and female WT mice in the same cage were carefully fed and closely observed for 9 weeks. At this time, ALS mice had shown progressive impairment of early motor function. We killed untreated mice at 9, 13, and 17 weeks of age, taking tissue samples.

**Fig. 1:**
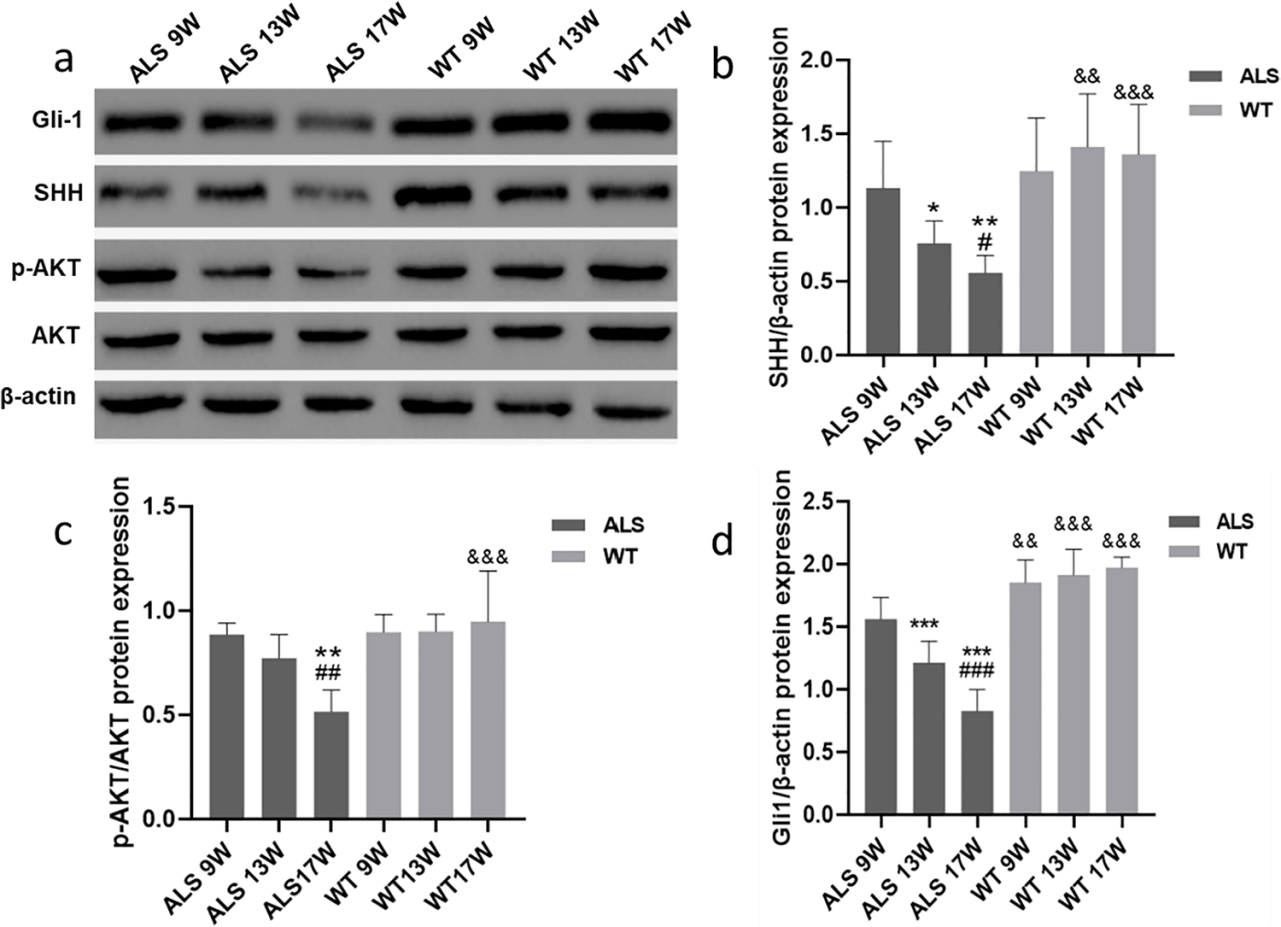
The expression of SHH and PI3K/AKT signaling pathway related proteins (**a**). The protein expressions of SHH, Gli1 and p-AKT (**b**, **c**, **d**). The protein level of lumbar spinal cord homogenate is 1 mg/ml. (*N* = 10: the number of animals analyzed per group, the same for the rest of the figures, *: vs ALS 9 W group, #: vs ALS 13 W group, &: vs ALS 17 W group, */#:*p* < 0.05, **/##/&&:*p* < 0.01, ***/###/&&&:*p* < 0.001)

**Fig. 2:**
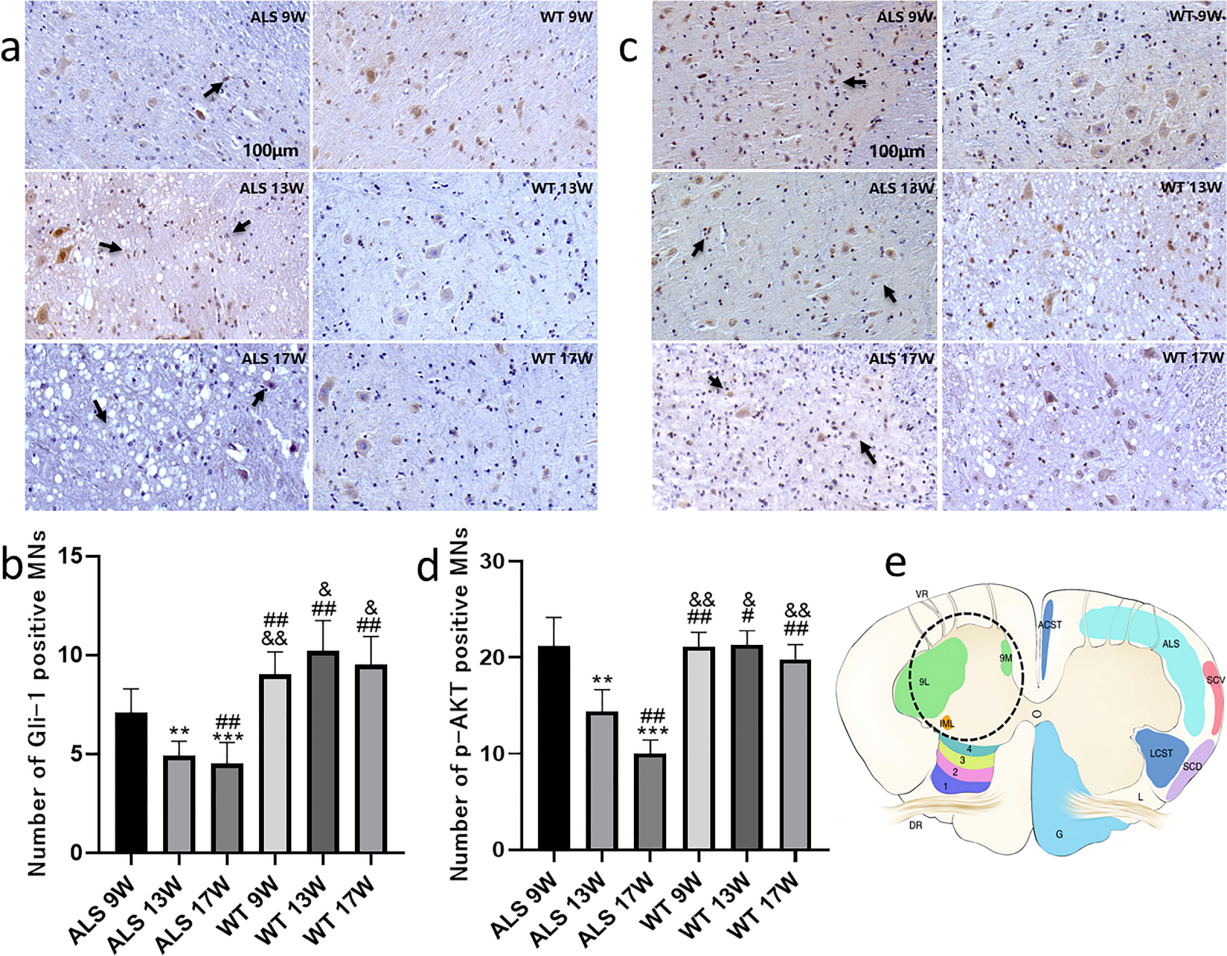
Immunohistochemical staining results of anterior horn of lumbar spinal cord in ALS mice. The number of Gli1 (**a**) and p-Akt (**c**) motor neuron cells in the anterior horn area gradually decreases with the increase of mouse age, and there are abnormalities such as cell atrophy and nuclear pyknosis (indicated by the arrow). The difference between the marked groups (**b** and **d**) was statistically significant. Samples of the spinal cord (**e**)-9L: lamina 9 – lateral motor column (extremities); 9 M: lamina 9 – medial motor column (axial muscles) [43]. (*N* = 10, *: vs ALS 9 W group, #: vs ALS 13 W group, &: vs ALS 17 W group, #: *p* < 0.05, **/##/&&: *P* < 0.01, ***: *p* < 0.001. Scale bar of all pictures: 100 μm)

There was no significant difference in SHH protein expression between ALS and WT mice at 9 weeks of age, as shown in Fig. 1a. However, at 13 and 17 weeks of age, the expression of SHH decreased with the age of ALS mice (*P* < 0.05) (Fig. 1b). At the same time, the expression of transcription factor Gli-1 downstream of the SHH pathway was also downregulated (*P* < 0.05) (Fig. 1c). With the progression of ALS disease, p-Akt protein expression also statistically significantly decreased (*P* < 0.05) (Fig. 1d). Through immunohistochemical staining, we observed that the motor neuron cells in the lumbar enlargement of the spinal cord increased express Gli1 in the anterior horn of gray matter compared to the posterior horn, posterior cord, and lateral cord of gray matter (Fig. 2a). In the spinal cord of WT mice, evenly distributed motor neuron cells with normal morphology were observed in the horizontal section of the spinal cord. Compared with WT mice, the overall number of motor neuron cells in the spinal cord of ALS mice decreased, and the motor neuron cells expressing Gli-1 (Fig. 2b) and p-AKT (Fig. 2d) in the anterior horn gradually reduced with the progression of the disease (*P* < 0.01).

### SHH Participates in the Development of ALS by Mediating PI3K/AKT Pathway

**Fig. 3:**
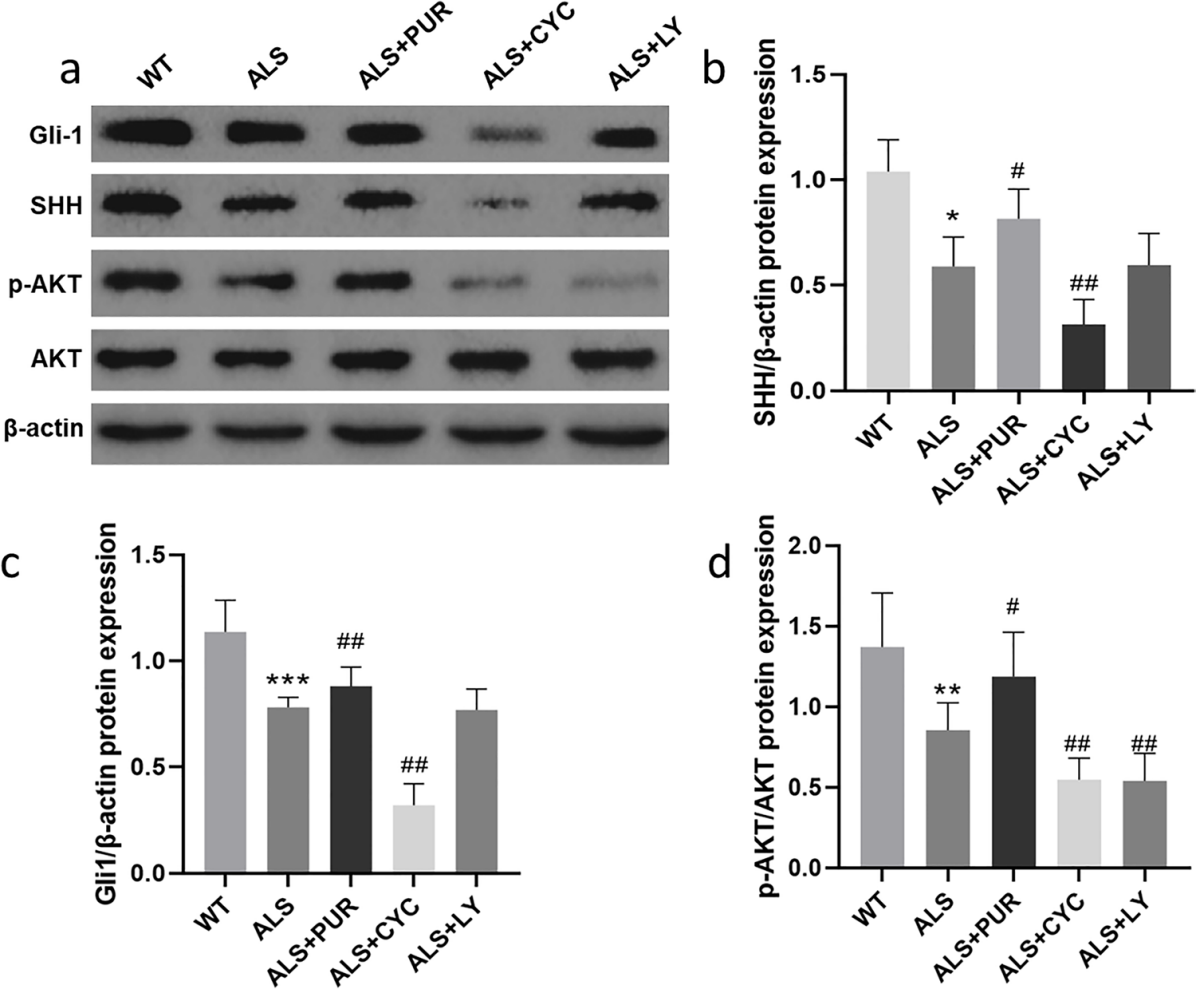
The expression of SHH and PI3K/AKT pathway related proteins (**a**). The expression of SHH, Gli1 and p-AKT (**b**, **c**, **d**) in ALS group, ALS + PUR group, ALS + CYC group and ALS + LY group. (*N* = 10, *: vs ALS group, #: vs ALS + PUR group, &: vs ALS + CYC group. */#/&: *p* < 0.05, **/##/&&: *p* < 0.01, ***/###: *p* < 0.001)

**Fig. 4:**
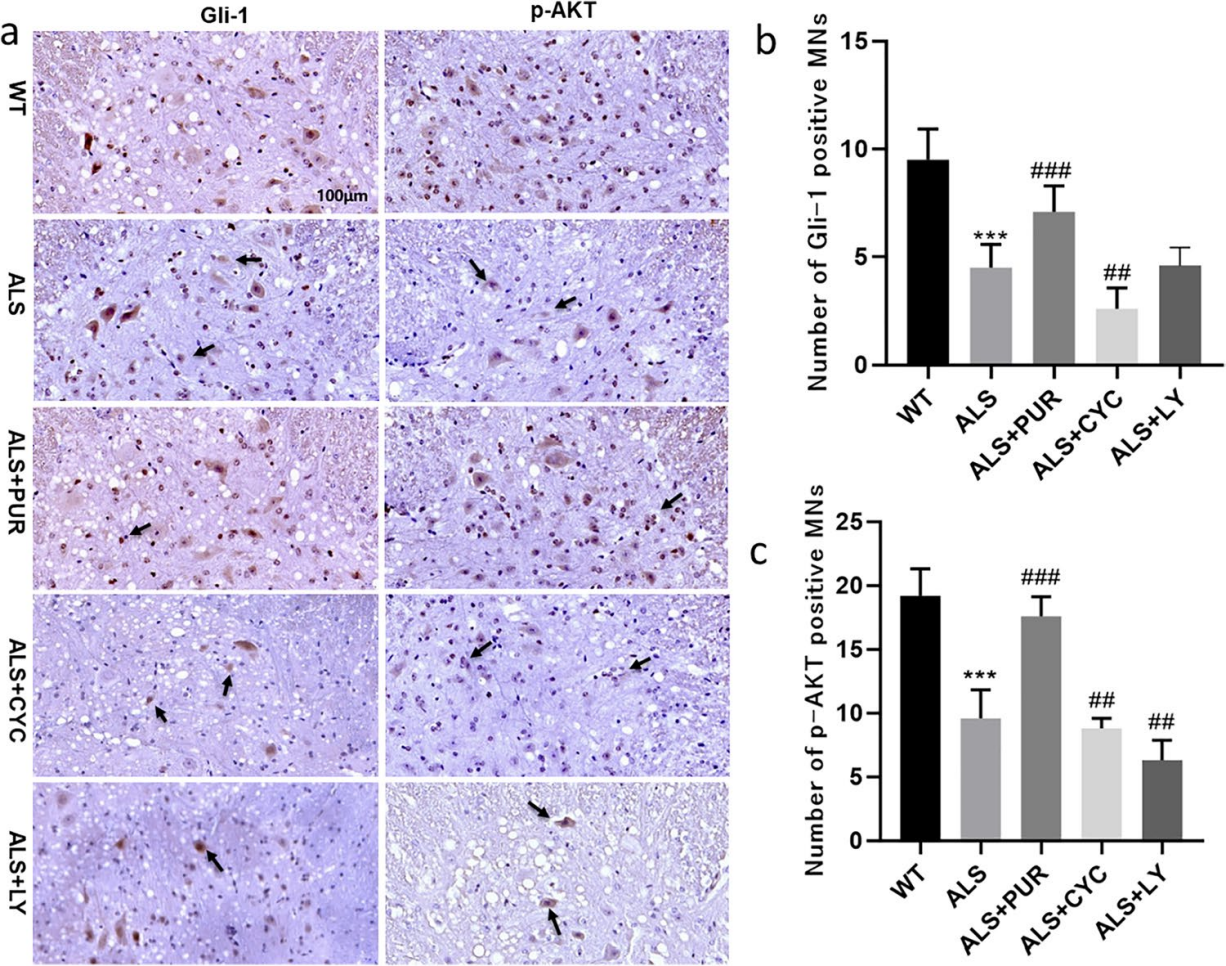
Immunohistochemical staining of horizontal sections of anterior horn of lumbar spinal cord in mice. Gli-1, p-AKT (**a**) moto neuron cells in the anterior horn area and the number of moto neuron cells (**b**, **c**), abnormal moto neuron cells (indicated by the arrow) can be seen. The difference between the groups of markers was statistically significant. (*N* = 10, *: vs WT group, #: vs ALS group. ##: *P* < 0.01, ***/###: *P* < 0.001. Scale bar of all pictures: 100 μm)

Female ALS mice with the apparent onset of disease (9 weeks old) were treated with different medicine, and the expression of Gli-1, SHH, and PI3K/AKT proteins was measured in Fig. 3a. Compared to WT mice, SHH, Gli-1, and p-AKT protein in the ALS group decreased; however, treatment with the PUR group improved these proteins' expression significantly. CYC-treated ALS mice considerably reduced SHH, Gli-1, and p-AKT proteins expression. There was no significant difference in SHH protein expression between ALS and ALS + LY mice (*P* < 0.05) (Fig. 3b). In addition, as can be seen in Fig. 3c, the expression of Gli-1 between ALS and ALS + LY mice were also the same. However, the expression of the p-AKT protein (Fig. 3d) in ALS + CYC and ALS + LY mice decreased statistically significantly compared to other groups (*P* < 0.05). The expression of Gli-1 protein and p-AKT protein in the lumbar spinal cord of ALS mice was detected by immunohistochemistry, and we counted the number of motor neuron cells under the microscope (Fig. 4a). Compared to the ALS mice (Fig. 4b), the numbers of Gli-1 cells increased in ALS + PUR mice while decreasing significantly in ALS + CYC mice; meanwhile, p-AKT cells behaved the same as Gli-1 cells in ALS + PUR mice as well as in ALS + CYC mice (Fig. 4c). Moreover, p-AKT cells in ALS + LY mice decreased significantly, while the number of Gli-1 cells in ALS + LY mice remained unchanged (*P* < 0.05) (Fig. 4b and c).

### PUR Prolonged the Survival Time of ALS Mice and Delayed the Development of ALS

Female ALS mice were randomly divided into ALS group, ALS + PUR group, and ALS + CYC group, with 10 experimental mice in each group. In previous experiments, we found that the SHH and the Gli-1 (downstream protein) expression decreased significantly in the lumbar spinal cord of ALS mice. Therefore, we used the receptor agonist PUR or antagonist CYC to intervene in the SHH pathway and observe its effects on mice's body weight and survival rate.

**Fig. 5:**
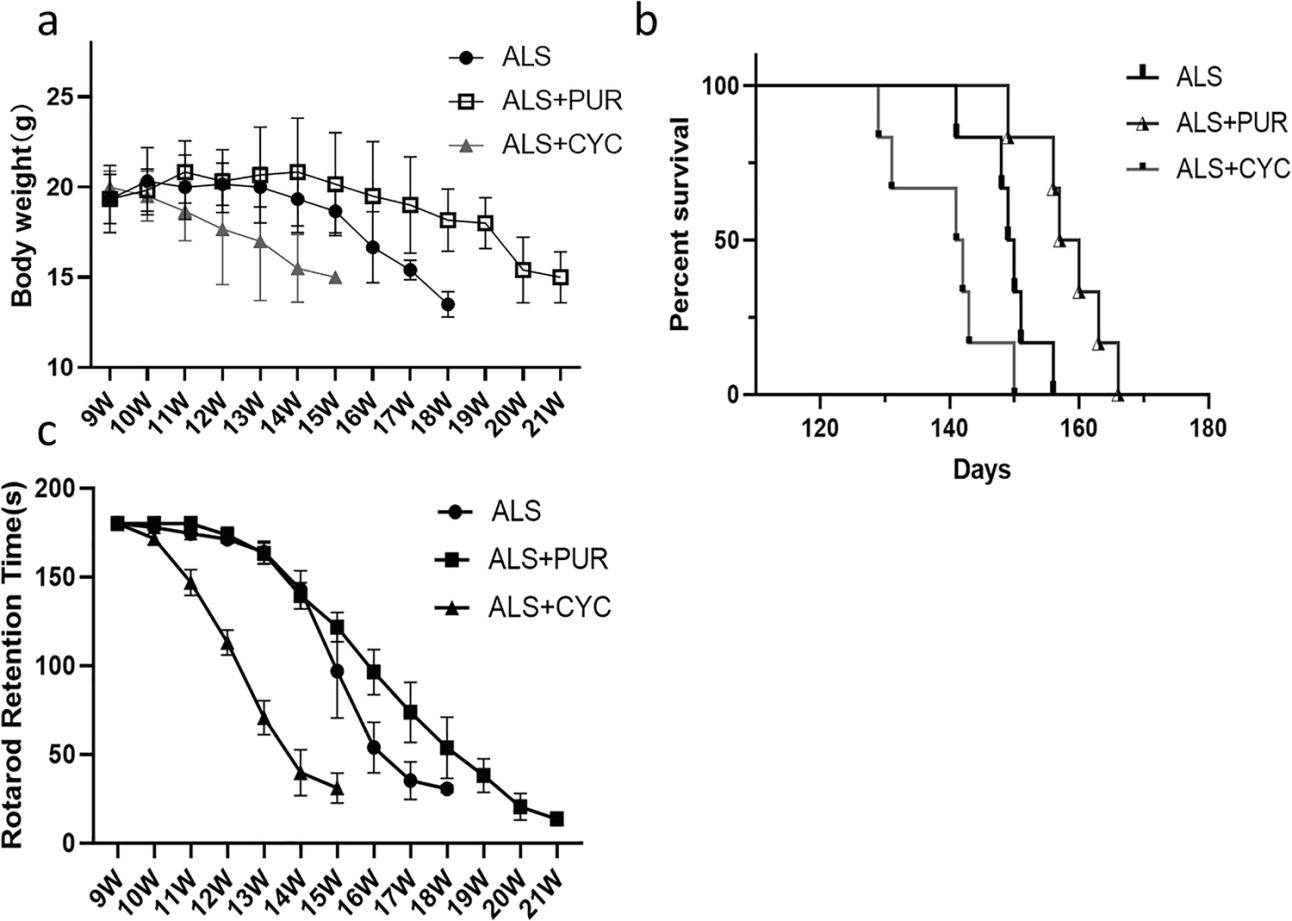
Changes in body weight of mice in ALS group, ALS + PUR group and ALS + CYC group (**a**) and their survival rate during this period (**b**). Recorded the time of mouse rotarod test (**c**) in each group (*N* = 6, #: vs ALS + CYC group, *: vs ALS + PUR group. ##: *P* < 0.01, ***: *P* < 0.001)

Compared with the mice in the ALS group, the weight of ALS + PUR group mice did not change significantly (P = 0.396, Fig. 5a), but the survival rate (P = 0.003, Fig. 5b) increased significantly, and the survival time prolonged. The weight of ALS + CYC group mice decreased. There was no statistical significance (P = 0.069). However, the survival time decreased significantly (*P* = 0.002). The rod rotation time of ALS mice gradually shortened from 9 weeks. The latency to fall of ALS + PUR group was declining slower than the ALS group, indicating PUR could delay the damage of motor function of ALS transgenic mice (P = 0.022) (Fig. 5c). The latency to fall of the CYC-treated mice was significantly shortened compared to ALS and ALS + PUR treated mice (*P* < 0.001).

### PUR Delays Muscle Morphological Damage in SOD1-G93A Mice

**Fig. 6:**
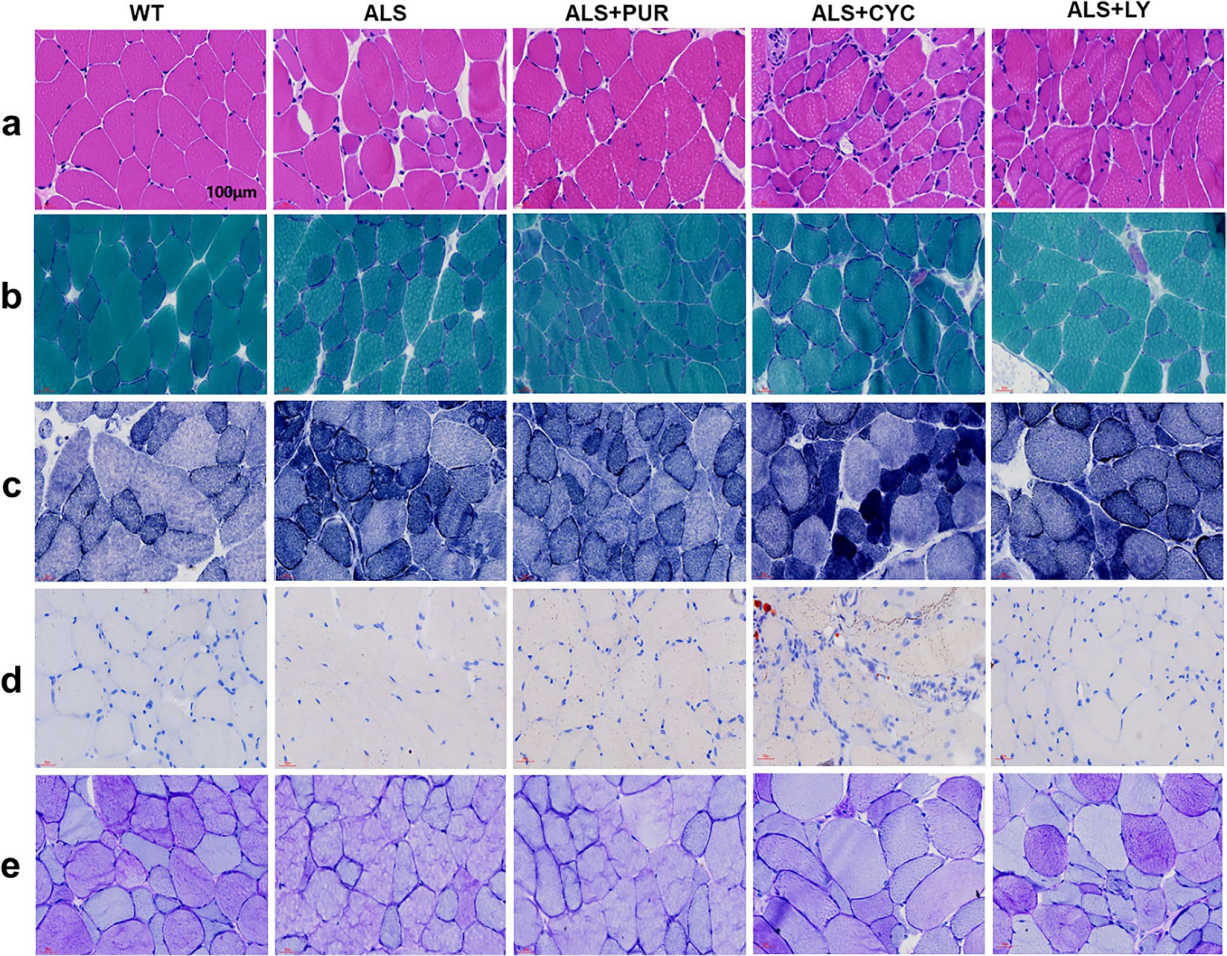
In different groups, H&E staining (**a**). MGT staining (**b**). NADH-TR staining (**c**). Oil red O staining (**d**). PAS staining (**e**). (Scale bar of all pictures: 100 μm)

Muscle atrophy is one of the main manifestations of ALS disease. We analyzed the morphological changes of the gastrocnemius muscle in order to confirm the beneficial effect of PUR on muscle tissue of ALS disease mice. We performed a histochemical analysis with H&E, MGT, NADH-TR, oil red O, and PAS staining. Compared to the WT animals, H&E staining showed that ALS disease animals had abnormal manifestations such as muscle cell atrophy and nuclear aggregation. The deterioration of muscle tissue was more severe in ALS + CYC and ALS + LY group animals, whereas herein the ALS + PUR animals, the deterioration was alleviated (Fig. 6a). The MGT staining can demonstrate ragged red fibers and intermyofibrillar red deposits. In normal cells (WT control group), the muscle fibers were dark green, and the nucleus and peripheral nerve myelin sheath were purplish-red (Fig. 6b).

Stained and broken red fibers can be observed under the sarcolemma in the ALS group, mainly composed of abnormally increased mitochondria. NADH-TR staining was located in mitochondria and sarcoplasmic reticulum. We used this method to observe the abnormal distribution of muscle fibers and myofibrillar reticulum (Fig. 6c). In the WT group, the normal types of muscle fibers: type I muscle fibers were dark blue, and type II muscle fibers were light blue and distributed in a mosaic shape. Due to sarcoplasmic condensation, deep staining in severely atrophic muscle fibers can also occur in NADH-TR staining. The number of atrophic cells stained with dark blue in the ALS group was more than that of the WT group. In addition, muscle fiber fragmentation occurred in the ALS + CYC group and ALS + LY group. The morphology deterioration of muscle fiber was reduced in the ALS + PUR group. Oil red O staining specifically shows neutral fat, which is deeply colored and can show smaller fat droplets (Fig. 6d). The results showed no noticeable abnormalities in each group, indicating no fat deposition (bright red or orange) in mice gastrocnemius muscle cells. The content and distribution of glycogen in cells can be observed with the PAS staining (Fig. 6e). In normal cells, type I muscle fibers are reddish, type II muscle fibers are purplish red, and the nucleus is blue. The abnormal myocyte in ALS mice showed glycogen loss. In ALS + CYC and ALS + LY mice, the glycogen loss was worse, and the glycogen was unevenly distributed in different muscle fibers.

## Discussion

SHH signaling pathway is an important signal transduction pathway related to the development of the central nervous system, which plays a key role in the differentiation of precursor cells of neurons in the body [43–45]. The Shh signaling pathway is incompletely understood and interactions with other signaling pathways are possible [47]. SHH may maintain neural stem cells and promote the formation of oligodendrocytes (Yang [48]. Oxidative stress is one of the most important pathological mechanisms in neurodegenerative diseases. SHH promoted the expression of the anti-apoptotic gene Bcl-2 and inhibited expression of pro-apoptotic gene Bax. Activation of SHH signals upregulated both Neurotrophic factors vascular endothelial growth factor (VEGF) and brain-derived neurotrophic factor (BDNF) [49]. SHH signaling pathway was involved in these diseases, but the underlying mechanisms remained elusive.

This study also found the damage to the SHH signal pathway in the spinal cord of ALS female mice and SHH expression by drug stimulation delayed the disease progression of spinal motor neurons in ALS mice. We observed the cell morphology, calculated the numbers of spinal cord tissue, and did Western blot proteins testing of different day-ages mice. The SHH, Gli-1, and p-AKT proteins expression were down-regulated in ALS mice, indicating that ALS mice's SHH pathway and PI3K/AKT signaling pathway are affected.

Gli is SHH downstream signal molecule relating to the central nervous system. Up-regulation of Crabp1 by SHH is mediated by Gli1, which binds the Gli target sequence in Crabp1’s neuron-specific regulatory region upstream of minimal promoter [50]. Activation of PI3K/AKT pathway and SHH/Gli1 pathway is associated with glioma progression [51]. CPH was neuroprotective by restoring neurobehavioral alterations and significantly reducing the elevated PI3K, AKT, and mTOR protein levels, and modulating the apoptotic markers in rat brain homogenate [52]. SHH can increase the activity of the PI3K/AKT pathway, inhibit the activity of GSK3, and then inhibit the degradation of Gli protein by the ubiquitin–proteasome system (Kanda [53]. This study also found that activating SHH signaling by intraperitoneal injection of PUR can improve the disease symptoms and prolong the life of ALS model mice. The results showed that activating the SHH signaling pathway with PUR increased the Gli-1 expression and could thereby overcome the inhibition of p-AKT protein. When CYC inhibited the SHH signaling pathway, the p-AKT protein expression was reduced, resulting in a deterioration of the ALS symptoms. In the behavioral test, the results also showed that PUR injection could improve the motor function of mice, while CYC injection worsened the motor function of mice. These results suggest that Akt is a downstream molecule of SHH, and activation of PI3K/Akt pathway may be necessary for the protective effect of SHH on motor neurons.

To confirm the relation between PI3K/AKT signaling pathway and SHH expression. We used an intraperitoneal injection of the PI3K/AKT inhibitor LY294002  [41, 54]. The results showed that the expression of p-AKT in the PI3K/AKT signaling pathway was inhibited. However, the expression of Gil-1 was not affected, suggesting that SHH-mediated Gil-1 expression was independent of PI3K activity. The neuroprotective effect of SHH disappeared when PI3K/AKT signaling pathway was inhibited. Blocking PI3K/Akt pathway more significantly inhibited motor neuron cell viability than blocking SHH. The results further demonstrated that the protective effect of SHH on motor neurons is realized through the activation of the PI3K/Akt pathway, which is necessary for SHH to reduce apoptosis and improve cell survival.

Both the MAPK/ERK and PI3K/Akt pathways are regulated by Shh and are required for its effects on adult myoblasts. However, The PI3K/Akt pathway mediates and is essential for Shh-stimulated muscle cell differentiation and is essential for this process, whereas MAPK/ERK is less important [55]. In this study, H&E staining showed that ALS animals had obvious abnormal manifestations in muscle tissue, such as muscle cell atrophy and nuclear aggregation. The deterioration of these morphologies was more serious in ALS + CYC and ALS + LY animals. However, the deterioration was alleviated in ALS + PUR animals. MGT staining showed that the number of atrophic cells stained with dark blue in ALS animals increased. In addition, muscle fiber fragmentation occurred in ALS + CYC and ALS + LY animals. While the deterioration of muscle fiber morphology was more reduced in ALS + PUR animals, Oil red O staining was adopted to observe the fat deposition (bright red or orange) in mouse gastrocnemius muscle cells. The results showed no abnormality in each group; PAS staining showed glycogen loss in the abnormal myocyte in ALS animals and improved lesions in ALS + PUR animals, but there was no statistical significance. The glycogen loss of ALS + CYC and ALS + LY animals was worse than that of other groups, and glycogen was unevenly distributed in different muscle fibers. The results of the cell, tissue, and behavioral studies all showed that SHH could regulate the PI3K/AKT signaling pathway, and the disturbance of the regulation leads to disease worsening.

In this study, the SHH signaling pathway in SOD1 mutation-induced ALS was analyzed and shown to play a prominent role in neuronal degeneration in ALS. SHH signaling pathway can regulate ALS disease by mediating PI3K/AKT signaling pathway, and interfering with this pathway can significantly alleviate the progression of ALS disease. This study thereby provides a potentially effective target for the treatment of ALS disease and provides primary theoretical data for further studying the molecular mechanism of motor neuron degeneration.

## Conclusion

SHH and PI3K/AKT signaling pathways inhibited in SOD1-G93A female mice.

The activation of the SHH signaling pathway can interfere with the expression of the PI3K/AKT signaling pathway.

The activation of the SHH-PI3K/AKT signaling pathway can protect motor neuron cells and improve motor function, thereby prolonging the life of mice suffering from ALS.

## Data Availability

The datasets generated during and/or analyzed during the current study are available from the corresponding author on reasonable request.
